# Genome-Wide Characterization of the *HSP20* Gene Family Identifies Potential Members Involved in Temperature Stress Response in Apple

**DOI:** 10.3389/fgene.2020.609184

**Published:** 2020-11-06

**Authors:** Fuwen Yao, Chunhui Song, Hongtao Wang, Shangwei Song, Jian Jiao, Miaomiao Wang, Xianbo Zheng, Tuanhui Bai

**Affiliations:** College of Horticulture, Henan Agricultural University, Zhengzhou, China

**Keywords:** apple, *HSP20* family, heat stress, genome-wide analysis, gene expression

## Abstract

Apple (*Malus domestica* Borkh.), an economically important tree fruit worldwide, frequently suffers from temperature stress during growth and development, which strongly affects the yield and quality. Heat shock protein 20 (*HSP20*) genes play crucial roles in protecting plants against abiotic stresses. However, they have not been systematically investigated in apple. In this study, we identified 41 *HSP20* genes in the apple ‘Golden Delicious’ genome. These genes were unequally distributed on 15 different chromosomes and were classified into 10 subfamilies based on phylogenetic analysis and predicted subcellular localization. Chromosome mapping and synteny analysis indicated that three pairs of apple *HSP20* genes were tandemly duplicated. Sequence analysis revealed that all apple *HSP20* proteins reflected high structure conservation and most apple *HSP20* genes (92.6%) possessed no introns, or only one intron. Numerous apple *HSP20* gene promoter sequences contained stress and hormone response *cis*-elements. Transcriptome analysis revealed that 35 of 41 apple *HSP20* genes were nearly unchanged or downregulated under normal temperature and cold stress, whereas these genes exhibited high-expression levels under heat stress. Subsequent qRT-PCR results showed that 12 of 29 selected apple *HSP20* genes were extremely up-regulated (more than 1,000-fold) after 4 h of heat stress. However, the heat-upregulated genes were barely expressed or downregulated in response to cold stress, which indicated their potential function in mediating the response of apple to heat stress. Taken together, these findings lay the foundation to functionally characterize *HSP20* genes to unravel their exact role in heat defense response in apple.

## Introduction

Temperature is an important factor affecting plant growth and geographical distribution (Wang et al., 2016). Most plants undergo optimal growth and development within a narrow temperature range and can only tolerate minor fluctuations. Fluctuations beyond optimal range result in temperature stress, which is one of the most severe environmental stresses affecting plant growth, development and survival worldwide (Peleg and Blumwald, 2011). High and low temperature stresses have rapid and severe effects on plant cell physiology, altering gene expression, protein levels, and energy consumption (Wang et al., 2017; Shen et al., 2019; Suzuki, 2019). Plants have developed a series of physiological and molecular strategies to overcome temperature stress over evolutionary time (Asea et al., 2016; Huo et al., 2020). Heat shock proteins (HSPs) are one of the strategies, and HSPs are essential in regulating growth, development and stress response in plants (Waters et al., 1996; Wang et al., 2004; Asea et al., 2016; He et al., 2019).

*HSPs* can be divided into five categories according to their molecular weight: *HSP100s*, *HSP90s*, *HSP70s*, *HSP60s*, and *HSP20s* (Waters, 2013; Zhao et al., 2018). Of these groups, *HSP20* is commonly associated with temperature stress in plants (Waters, 2013). As genomes for more species are sequenced, the *HSP20* gene family has been identified in various plants. Nineteen *HSP20* genes have been identified in *Arabidopsis* (Scharf et al., 2001), 39 in rice (Ouyang et al., 2009), 42 in tomato (Yu et al., 2016), 44 in watermelon (He et al., 2019), and 48 in grape (Ji et al., 2019). Previous studies have suggested that *HSP20* genes are involved in regulating a diverse array of developmental processes and responses to abiotic stresses, especially in heat stress (Guo et al., 2015; He et al., 2019; Ji et al., 2019). Yu et al. (2016) identified tomato *HSP20* family genes and analyzed their functions in abiotic-stress responses. Most pepper *HSP20* genes were highly induced by heat stress (Guo et al., 2015). Among the *GmHSP20* genes, five were shown to be involved in the soybean response to cold stress (Lopes-Caitar et al., 2013). Interestingly, the same *HSP20* genes exhibited a different expression pattern in the heat tolerant and sensitive plants. These differences in expression pattern indicate the roles of *HSP20* in heat tolerance. In addition, some studies have further verified the role of *HSP20s i*n stress tolerance using transgenic methods. For example, overexpressing with *WsHSP26* in *Arabidopsis* showed improved heat tolerance (Mu et al., 2013). Similarly, transgenic rice over-expressing *OsHSP17.7* conferred enhanced tolerance to heat stress (Murakami et al., 2004). Together, these studies reveal the crucial role of *HSP20* genes in mediating temperature stress tolerance.

Apple (*Malus domestica* Borkh.), an economically important fruit crop, is widely planted in temperate zones (Dobránszki and Teixeira da Silva, 2010). However, apple trees frequently suffer from both high and low temperature stresses during their life cycle, which strongly affect apple quality and yield. After suffering continuous heat stress in summer, the leaf and fruit of apple can be severely damaged; resulting in tissue discoloration and sunburn of the fruit surface (Torres et al., 2017). It is reported that fruit sunburn causes 10–40% yield losses in all major apple growing regions around the world (Wang et al., 2020). After suffering cold stress in early spring, the pollination, new leaves and shoots of apple can be severely damaged, thereby greatly reducing the yield and quality of apple. The entire genome of apple has been sequenced, providing powerful resource for the mining and identification of *HSP20* gene family members at the whole genome level.

In the present study, we identified *HSP20* genes from the apple genome using bioinformatics methods, and determined their chromosomal locations, gene duplication, phylogenetic relationships, gene structures, and conserved domains, as well as *cis*-elements. Furthermore, we analyzed the expression patterns of the apple *HSP20* genes using qRT-PCR in order to determine their roles in response to heat and cold stresses. Our findings provide valuable information for subsequent research on the functions and regulatory mechanisms of potentially important *HSP20* genes that are crucial in modulating heat stress tolerance in apple.

## Materials and Methods

### Plant Materials and Stress Treatments

Seeds of *M. hupehensis* were stratified using sand at 4°C for 40 days, as previously described (Bai et al., 2013). Then, one germinated seed was planted in one plastic pot (6 cm × 6 cm) filled with soil/organic substrate (1:5, v: v) in a greenhouse under natural light, 22–28°C (day) and 5–10°C (night), and a relative humidity of 60–70%. After growing for 70 days, uniform seedlings were randomly divided into three groups and transferred to different chambers for temperature stress: (1) control (CK), growth chamber were maintained at 25 ± 1°C, 16-h photoperiod (160 μmol m^–2^ s^–1^) and relative humidity of 60–70%; (2) heat stress (HS), growth chamber maintained at 40 ± 1°C; (3) cold stress (CS), growth chamber maintained at 4 ± 1°C, 16-h photoperiod (160 μmol m^–2^ s^–1^) and relative humidity of 60–70%. Each treatment contained three biological replicates and 30 plants for each replicate. At 0, 4, 8, 12, and 24 h after treatments, the samples were rapidly frozen in liquid nitrogen and stored at −80°C until RNA extraction.

### Genome-Wide Identification of the *HSP20* Genes in Apple

The reference apple genome and protein sequences were downloaded from the Genome Database for Rosaceae (GDR^1^). The apple *HSP20* candidates with an e-value ≤ 0.001 were identified based on the Hidden Markov Model (HMM) profile (PF00011) downloaded from Pfam protein family database^2^. The SMART database^3^ was used to further confirm the conserved *HSP20* gene domain. ProtParam^4^ was used to predict the potential chemical characteristics of the *HSP20* genes. ProtComp^5^ was used to predict the subcellular localization.

### Phylogenetic Analysis and Classification of Apple *HSP20* Genes

The full-length amino acid sequences of *HSP20* genes derived from *Arabidopsis* and rice downloaded from the Ensembl Plants Database^6^ were combined with newly identified *HSP20* genes in apple and used for phylogenetic analysis. The phylogenetic tree was constructed using MEGA 6.0^7^.

### Structure and Domain Analysis of Apple *HSP20* Genes

The structures of *HSP20* genes were identified using TBtools software (Chen et al., 2018). The conserved motifs of *HSP20* genes were identified using MEME Suite 5.1.1^8^, and the parameters were as follows: optimum motif width ranges from 6 to 200 amino acid residues and maximum of 10 misfits. The upstream 2.0 kb promoter sequence of the apple *HSP20* genes was downloaded from the GDR and submitted to PlantCARE^9^ to identify the *cis*-elements (Lescot et al., 2002).

### Chromosomal Location and Synteny Analysis

All identified *HSP20* genes were mapped to apple chromosomes using TBtools software based on the information available at GDR. Synteny analysis of *HSP20* genes was conducted using Circos v. 0.63^10^.

### RNA Extraction, cDNA Library Construction, and Sequencing

Total RNA was extracted from the leaf tissues according to the CTAB method (Chang et al., 1993). Each sample was 0.5 g and three biological replicates were performed. RNA concentrations were determined using a NanoDrop 1000 (Thermo Fisher Scientific, Waltham, MA, United States) and quality was assayed on a 1% agarose gel. The sample libraries were prepared according to the RNA-Seq library constructed flow path and sequenced on an Illumina HiSeq 4000 system. The raw sequence data from the sequence was used for analysis. After filtering the low quality reads and contaminant sequences, the clean reads were aligned to the apple genome (GDDH13 Version 1.1^11^) (Daccord et al., 2017) using HISAT2 (Kim et al., 2015). Stringtie software was used to assemble the transcript (Pertea et al., 2016). Gene expression was calculated using the fragments per kilobase of transcript per million (FPKM) fragments mapped Reads method (Mortazavi et al., 2008). TBtools was used to generate the heatmap. The RNA-seq data were available at NCBI^12^.

### qRT-PCR Analysis

Quantitative real-time PCR (qRT-PCR) was used to analyze the gene expression. Primers (Supplementary Table S1) were designed to amplify products of 150–250 bp using Primer 5.0 software. qRT-PCR was performed using ABI-7500 Connect Real-Time PCR Detection System. cDNAs were diluted to 200 ng and run in three technical replicates, with 1 μL template in a reaction volume of 20 μL. PCR amplification conditions were as follows: 95°C for 5 min for initial denaturation, then 45 cycles of 94°C for 20 s, 55°C for 20 s, and 72°C for 10 s. Fluorescence was measured at the end of each cycle. A melting curve analysis was performed to determine whether a single product was amplified. The apple Actin gene was used as an internal standard in the analysis. The relative expression level of each gene was calculated according to the 2^–ΔΔCT^ method (Livak and Schmittgen, 2001). Values for mean expression and standard error (SE) were calculated from the results of three independent biological replicates.

## Results

### Genome-Wide Identification of *HSP20* Genes in Apple

A total of 45 *HSP20* protein sequences were found in the apple cultivar “Golden Delicious” genome. Among 45 *HSP20* sequences, four sequences lacked the conserved domain. Ultimately, 41 sequences were identified as genes in the apple *HSP20* family and named *HSP20-1* to *HSP20-41* based on the position of the genes on the chromosomes (Supplementary Table S2). Gene name, gene ID, chromosomal location, open reading frame (ORF), amino acid (AA), molecular weight (MW) and isoelectric point (pI) for each gene are in Table 1. Sequence analysis showed that these *HSP20* proteins vary widely in length, from 88 (*HSP20-32*) to 363 (*HSP20-4*) AAs, and predicted MWs from 9.98 kDa (*HSP20-32*) to 39.47 kDa (*HSP20-4*). The ORF lengths of the *HSP20* genes ranged from 267 bp (*HSP20-32*) to 1,092 bp (*HSP20-4*), and the predicted pI-values of *HSP20* proteins ranged from 4.60 (*HSP20-5*) to 9.85 (*HSP20-10*).

**TABLE 1:** Characteristics of *HSP20* genes identified in apple.

Gene name	Sequence ID	Chr	Genomic location	ORF (bp)	AA	MW (kDa)	pI	Subcellular localization
*HSP20-1*	MD00G1181700	0	42820094-42820435	342	113	12.93	9.49	Cytoplasm
*HSP20-2*	MD01G1144400	1	25475100-25479690	810	269	30.82	9.38	Chloroplast
*HSP20-3*	MD01G1227600	1	31728955-31730100	480	159	18.05	5.94	Chloroplast
*HSP20-4*	MD02G1259500	2	31287370-31288929	1092	363	39.47	9.53	Chloroplast
*HSP20-5*	MD03G1081800	3	6580874-6581326	453	150	16.75	4.60	Chloroplast
*HSP20-6*	MD04G1140600	4	22893411-22893881	471	156	18.17	6.87	Cytoplasm
*HSP20-7*	MD05G1183400	5	31196823-31197443	621	206	23.14	6.46	Chloroplast
*HSP20-8*	MD05G1240300	5	37103604-37104083	480	159	18.32	5.72	Cytoplasm
*HSP20-9*	MD05G1343700	5	46361886-46362915	414	137	16.11	5.86	Chloroplast
*HSP20-10*	MD06G1060300	6	9340096-9341581	789	262	29.46	9.85	Nucleus
*HSP20-11*	MD06G1188500	6	32557693-32558803	717	238	26.43	5.82	Chloroplast
*HSP20-12*	MD07G1210400	7	28942672-28943139	468	155	17.53	5.99	Cytoplasm
*HSP20-13*	MD07G1210700	7	28969132-28969626	468	155	17.55	5.39	Cytoplasm
*HSP20-14*	MD07G1210800	7	28970024-28970494	471	156	17.86	7.08	Cytoplasm
*HSP20-15*	MD07G1253800	7	32097833-32098492	660	219	24.43	6.99	Chloroplast
*HSP20-16*	MD07G1298000	7	35766162-35768343	672	223	24.68	7.87	Chloroplast
*HSP20-17*	MD08G1068000	8	5407860-5408476	465	154	17.10	6.61	Cytoplasm
*HSP20-18*	MD08G1068200	8	5414896-5415389	471	156	17.41	5.93	Cytoplasm
*HSP20-19*	MD08G1068300	8	5433838-5434323	486	161	17.84	4.77	Cytoplasm
*HSP20-20*	MD08G1068500	8	5449001-5449663	663	220	24.25	5.66	Chloroplast
*HSP20-21*	MD08G1068700	8	5470111-5470842	732	243	26.17	6.24	Chloroplast
*HSP20-22*	MD08G1068800	8	5474013-5474495	483	160	17.90	6.41	Cytoplasm
*HSP20-23*	MD08G1249100	8	31349994-31350611	618	205	22.94	7.08	Chloroplast
*HSP20-24*	MD09G1271100	9	34531591-34533232	438	145	16.36	6.92	Cytoplasm
*HSP20-25*	MD10G1171200	10	26409043-26409660	618	205	23.37	6.93	Endoplasmic reticulum
*HSP20-26*	MD10G1218300	10	31639809-31640195	387	128	14.87	5.94	Chloroplast
*HSP20-27*	MD10G1319400	10	40169759-40170934	411	136	15.88	5.39	Chloroplast
*HSP20-28*	MD11G1087100	11	7308309-7308791	483	160	18.21	5.81	Cytoplasm
*HSP20-29*	MD11G1088300	11	7365514-7366036	483	160	17.98	5.34	Cytoplasm
*HSP20-30*	MD11G1089300	11	7421465-7421947	483	160	18.22	5.39	Cytoplasm
*HSP20-31*	MD13G1108500	13	7793385-7800082	723	240	26.81	9.16	Chloroplast
*HSP20-32*	MD13G1120200	13	8835673-8835939	267	88	9.98	4.86	Cytoplasm
*HSP20-33*	MD15G1053500	15	3658892-3659347	456	151	16.79	9.09	Cytoplasm
*HSP20-34*	MD15G1053600	15	3662890-3663356	402	133	15.14	6.15	Chloroplast
*HSP20-35*	MD15G1053800	15	3675367-3676018	471	156	17.44	5.57	Cytoplasm
*HSP20-36*	MD15G1443700	15	54408605-54409228	624	207	23.17	6.31	Chloroplast
*HSP20-37*	MD16G1108600	16	7581626-7584321	711	236	26.71	9.36	Nucleus
*HSP20-38*	MD17G1020300	17	1535600-1536025	426	141	16.40	7.83	Cytoplasm
*HSP20-39*	MD17G1151000	17	13922992-13924497	471	156	17.36	7.72	Chloroplast
*HSP20-40*	MD17G1209800	17	25460073-25461508	732	243	27.26	8.45	Chloroplast
*HSP20-41*	MD17G1269200	17	32927931-32928852	339	112	12.53	6.30	Cytoplasm

*ORF, open reading frame; AA, amino acid; MW, molecular weight; pI, isoelectric point.*

### Phylogenetic Relationships of Plant *HSP20* Genes

**FIGURE 1:**
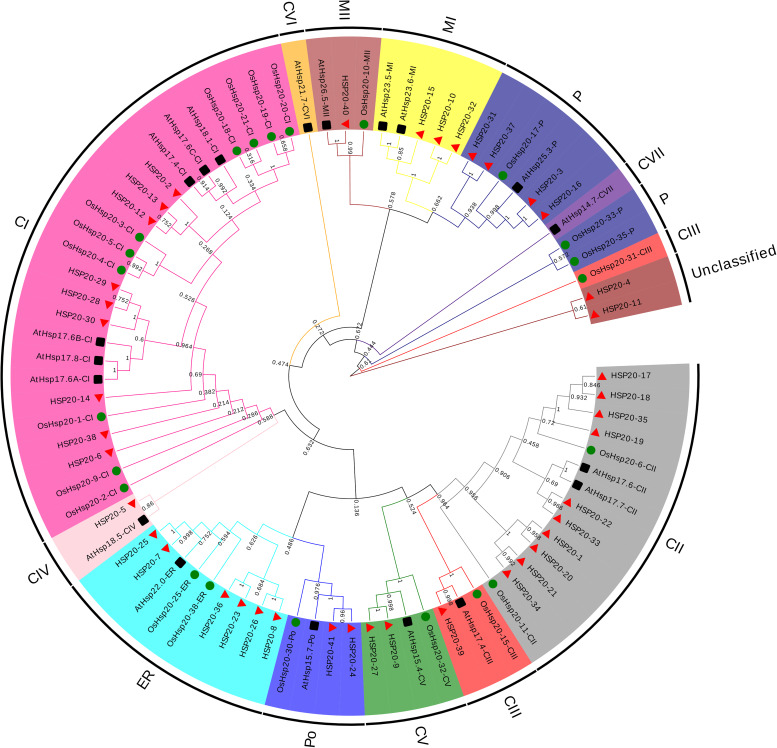
Phylogenetic analysis of HSP20 proteins from *Arabidopsis*, rice and apple. The phylogenetic tree was constructed using MEGA 6.0 based on the neighbor joining method with 1000 bootstrap replicates. The 12 subfamilies were distinguished with different colored arcs.

A phylogenetic tree was constructed based on the amino acid sequences of *HSP20* genes (Figure 1), 19 from *Arabidopsis*, 22 from rice (*Oryza sativa*) and 41 from apple (*M. domestica*) (Supplementary Table S3). The 82 *HSP20s* were divided into 12 subfamilies, 25 cytosol Is (CIs), 14 CIIs, 4 CIIIs, 2 CIVs, 4 CVs, 1 CVI, 1 CVII, five mitochondria Is (MIs), three MIIs, four peroxisomes (Pos), 9 from the endoplasmic reticulum (ER), and 8 plastids (Ps) based on the phylogeny and the subcellular localization. However, two apple *HSP20* genes (*HSP20-4* and *HSP20-11*) failed to cluster into any subfamily and were thus unclassified. Of the 12 subfamilies, 10 (CIs, CIIs, CIIIs, CIVs, CVs, MIs, MIIs, Pos, ER, and Ps) contained apple *HSP20* proteins. Except for the two unclassified *HSP20s*, 23 (59%) *HSP20s* were classified into CI-CVI, indicating that cytoplasm could be the primary functional area of the *HSP20* family in apple.

### Conserved Motifs and Gene Structure of *HSP20* Genes

**FIGURE 2:**
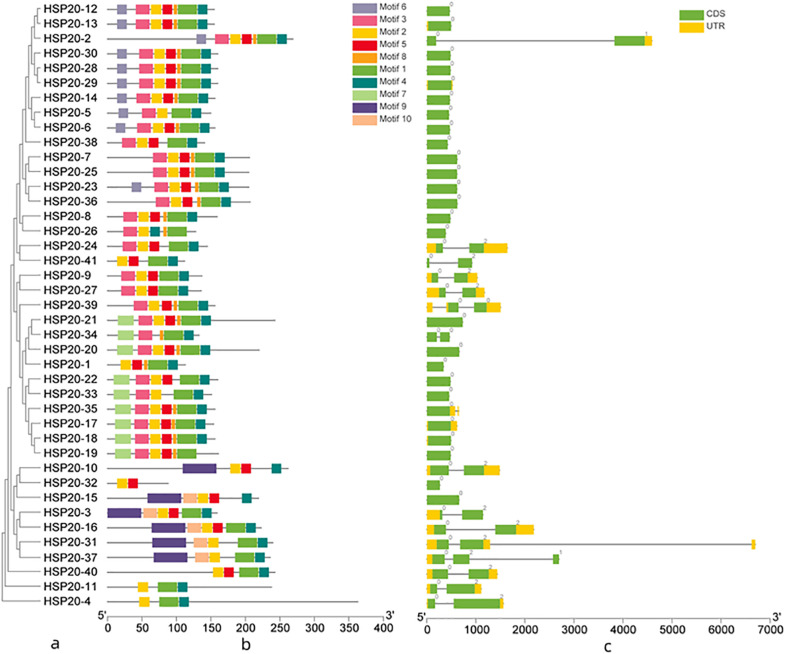
Phylogenetic tree, gene structure and domain analyses of HSP20s. **(a)** Phylogenetic tree of *HSP20s*. **(b)** Domain analyses of *HSP20* proteins. **(c)** Gene structure of *HSP20* genes. CDS sequences are represented with yellow round-corner rectangles and introns with gray lines, UTRs are shown with green boxes. Different colored boxes represent the different types of motifs.

The conserved motifs of apple *HSP20* gene family were identified and divided into 10 motifs (Figure 2b). The lengths of the 10 motifs ranged from 6 to 50 AAs, with the longest motif (9) containing 50 AAs and the shortest motif (8) containing six AAs; motifs 4, motifs 5, and motifs 6 have 15 AAs (Supplementary Table S4). The number of the conserved motifs for each *HSP20* gene ranged from 2 to 7. Most apple *HSP20* genes had two to seven conserved motifs, however, *HSP20-32* only contained two conserved motifs. The results suggested that the *HSP20* genes exhibited extreme divergence during the evolutionary process.

To gain insight into the evolutionary relationships of apple *HSP20* genes, the exon–intron structure of the *HSP20* genes was analyzed (Figure 2c). Among the *HSP20s*, 25 (60.9%) were intronless, 13 (31.7%) possessed one intron, and three genes (7.3%) – *HSP20-31*, *HSP20-37*, and *HSP20-38* – possessed two introns. Most *HSP20* genes thus have no introns or only one intron, suggesting relatively simple gene structures. Gene structure analysis showed that the genes with similar exon–intron patterns were grouped in the same cluster (Figure 2a).

### Chromosomal Location, Gene Duplication, and Synteny Analysis

**FIGURE 3:**
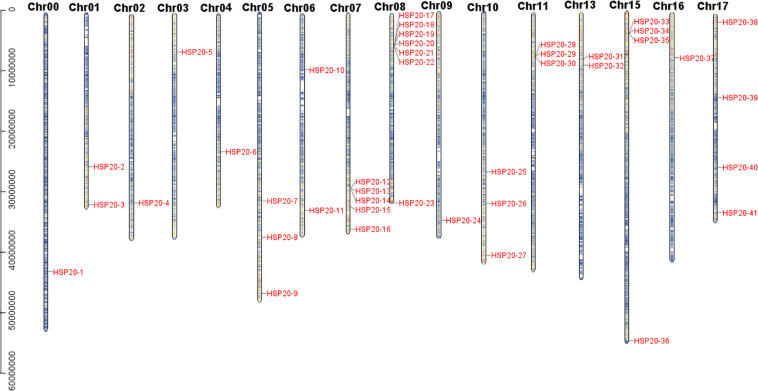
Distribution of *HSP20* genes in apple chromosomes. Forty-one *HSP20* genes was mapped to the 16 linkage groups (Chr 01 through Chr 17, except Chr 12 and Chr 14), whereas one *HSP20* gene were mapped on apseudo-chromosome, designated as Chr00.

**FIGURE 4:**
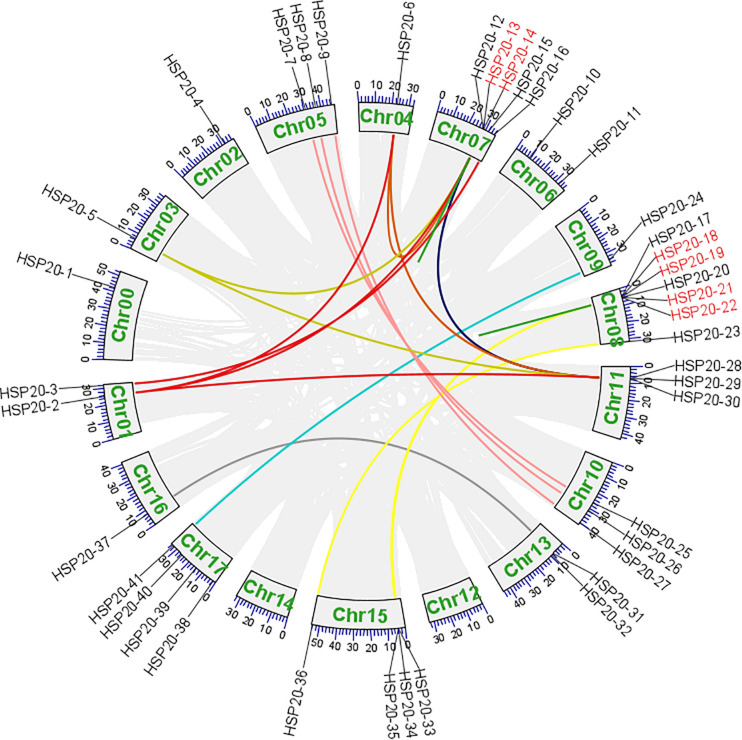
Syntenic relationships and gene duplications of the apple *HSP20* genes. The segmental duplicated genes are indicated by differently colored lines and tandem duplicated genes are indicated by red gene names.

A total of 41 apple *HSP20* genes were mapped on 15 chromosomes (Chr), except Chr 12 and 14, with an obviously non-uniform distribution (Figure 3). One *HSP20* gene (*HSP20-1*) could not be mapped on any of the apple chromosomes, so we mapped it on a pseudo-chromosome, named Chr00. In addition, most of the apple *HSP20* genes were located on the distal ends of the chromosomes. The biggest cluster was seven *HSP20* genes together on Chr 8, whereas the fewest *HSP20s* were found on Chrs 0, 2, 3, 4, 9, and 12 (one per Chr). Moreover, we analyzed the duplication events of apple *HSP20* genes (Figure 4). In total, 37 (90.2%) *HSP20* genes in apple exhibited segmental or tandem duplication. Twenty were segmentally duplicated and three pairs of genes (*HSP20-13* and *HSP20-14*, *HSP20-18* and *HSP20-19*, *HSP20-21*, and *HSP20-22*) were regarded as tandemly-duplicated genes (Supplementary Table S5). Chr 7 had the most duplication events, which could partly explain the larger numbers of *HSP20* genes on Chr 7, while Chr 0 and 12 did not contain any duplicated genes.

### Analysis of *Cis*-Element in Apple *HSP20* Gene Promoters

**FIGURE 5:**
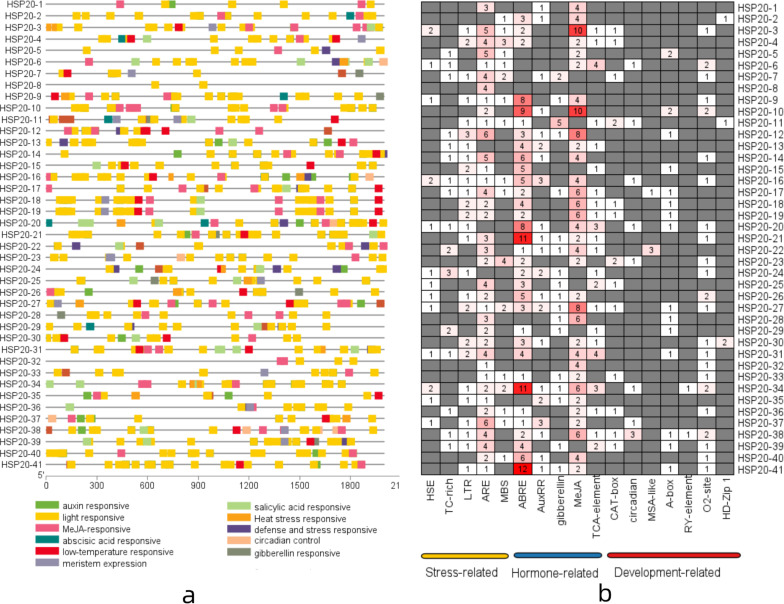
*Cis*-Element analysis of apple *HSP20* gene promoters. **(a)** The different colored blocks represent the different types of *cis-*acting elements and their locations in each *HSP20* gene. **(b)** The number of each *cis-*acting element in the promoter region of each apple *HSP20* gene.

To further investigate the potential regulatory mechanisms of the apple *HSP20* genes in response to temperature stress, the promoter in the upstream 2 kb region of 41 *HSP20* genes was analyzed to detect the *cis*-regulatory element. The results showed that three categories of *cis-*elements, including stress-related (heat, defense and stress, low-temperature and light), hormone-related (abscisic acid, auxin, gibberellin, MeJa, and salicylic acid), and plant development-related *cis-*elements (meristem expression and circadian control), were identified (Figure 5a). Among the stress-related *cis-*elements, 13 apple *HSP20* genes had the heat response elements (HRE) in their promoter regions, 26 apple *HSP20* genes had the low temperature response elements (LTR), which suggests a potential stress response under temperature conditions (Figure 5b). Among the hormone-related *cis*-elements, abscisic acid responsive (ABRE), salicylic acid responsiveness (TCA-element), auxin responsive (AUXRR-core), and MeJA-responsiveness (CGTCA-motif) were identified in the promoters of apple *HSP20* genes. All HSP20 genes contained light signal response elements, which indicate that *HSP20s* are essential in plant growth. The results indicate that the *HSP20* gene family is not only involved in the stress response, but is also involved in other physiological response processes.

### Expression Patterns of *HSP20s* in Response to Cold and Heat Stress

**FIGURE 6:**
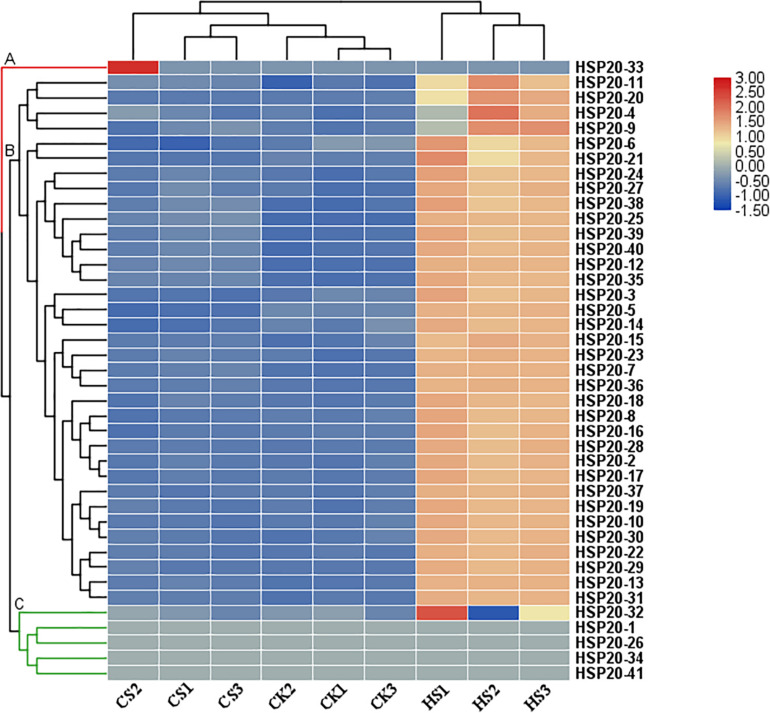
Heat map of the expression profiles of 41 apple *HSP20* genes in response to heat and cold stresses based on RNA-Seq data. Log2 based FPKM value was used to create the heat map with clustering. The color scale representing the relative expression values is shown on the left. CK, control, plants were maintained at 25 ± 1°C; HS, heat stress, plants were maintained at 40 ± 1°C; CS, cold stress, plants were maintained at 4 ± 1°C.

For a preliminary investigation of the functions of apple *HSP20* genes in response to heat and cold stress, nine RNA-seq libraries, including three independent biological replicates for the control, cold-treated and heat-treated, were constructed and sequenced. A heatmap of 41 apple *HSP20* genes was constructed using FPKM values from RNA-Seq data to estimate the expression levels of these genes (Figure 6). The heat map showed that the 41 *HSP20* genes clustered in three groups. Cluster A contains one member (*HSP20-33*) of 41 detectable *HSP20* genes, which was barely expressed after heat and cold stress treatment compared with the control. We found *HSP20-33* has no HREs in promoter region. This may be why the gene does not respond to heat stress. All 35 members from cluster B were mainly upregulated after 4 h of heat stress. However, these genes were nearly unchanged or downregulated under cold treatment. Cluster C contains five members, which had similar expression with cluster A, which was barely expressed after heat and cold stress treatment compared with the control.

**FIGURE 7:**
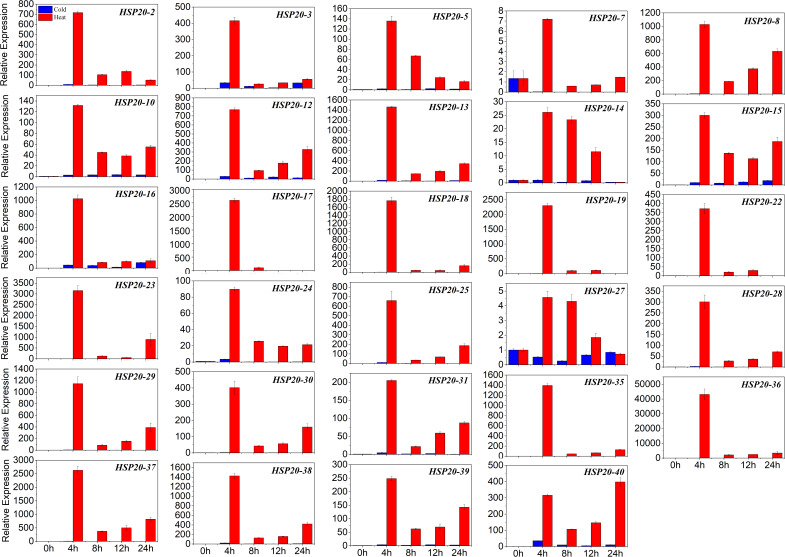
The relative expression levels of apple *HSP20* genes under heat and cold stresses. Mean expression value was calculated from three independent replicates. Vertical bars indicate standard deviation from three independent technical replicates.

To further identify which of these *HSP20* genes are most important in mediating heat and cold stress tolerances, 29 differentially expressed *HSP20* genes were selected to be further validated by qRT-PCR based RNA-Seq analysis (Figure 7 and Supplementary Table S6). Consistent with the RNA-Seq data, all 29 selected *HSP20* genes were up-regulated under heat stress. The expression levels of 12 *HSP20* genes (*HSP20-8, 13, 16, 17, 18, 19, 23, 29, 35, 36, 37*, and *38*) were extremely up-regulated (more than 1,000-fold) after 4 h of heat stress. Under heat stress, 29 *HSP2*0 genes were similarly expressed over time, with peak expression levels at 4 h, except for *HSP20-40*, with peak expression levels at 24 h, while most *HSP20* (*HSP20-2, 5, 7, 13, 17, 18, 19, 22, 23, 25, 28, 29, 30, 31, 35, 36, 37*, and *38*) genes were barely expressed in response to cold stress. However, eight apple HSP20 genes (*HSP20-3, 10, 12, 14, 15, 16, 27*, and *40*) were up-regulated under cold stress.

## Discussion

*HSP20s* are considered to be the most abundant plant stress responsive class among HSPs (Waters, 2013). They have been identified in potato, pepper, tomato, and soybean in responding to temperature stress (Lopes-Caitar et al., 2013; Guo et al., 2015; Yu et al., 2016; Zhao et al., 2018). But not studies have conducted an overall identification and characterization of the apple *HSP20* genes. Completion of high-quality apple genome sequencing has provided an opportunity to identify and characterize *HSP20* genes at the whole-genome level.

In the present study, we identified 41 *HSP20* genes and investigated their characteristics from the apple genome database. The number of apple *HSP20* genes was higher than that of *Arabidopsis* (19) (Scharf et al., 2001), slightly higher than rice (39) (Ouyang et al., 2009) but lower than that of watermelon (44) (He et al., 2019), potato (48) (Zhao et al., 2018), and grape (48) (Ji et al., 2019). This difference is most likely due to the fact that apple had gene duplications during evolution (Velasco et al., 2010; Ma et al., 2018). Gene duplication was reported to play an important role in the expansion of the number of gene families in plants (Blanc and Wolfe, 2004; Han et al., 2011). In the current study, 41 apple *HSP20* genes were unevenly mapped on 15 Chrs and most of the *HSP20* genes were located on the distal ends of the Chrs, which might contribute to the occurrence of duplication events in the apple *HSP20* gene family. We confirmed many tandem and segmental duplications in apple *HSP20* genes: 37 of 41 *HSP20* genes were affected by gene duplication, 20 of which were segmental duplication and 17 gene clusters were from tandem duplication. Ma et al. (2018) and Zuo et al. (2018) also found many tandem and segmental duplications in apple receptor-like kinase1-like kinase (CrRLK1L) genes and malate dehydrogenase (MDH) genes, respectively.

To determine the evolutionary relationships of *HSP20* genes, a phylogenetic tree was constructed based on the amino acid sequences of apple, *Arabidopsis* and rice *HSP20* genes. The phylogenetic analysis indicated that the apple *HSP20* family could be divided into 10 subfamilies (CIs, CIIs, CIIIs, CIVs, CVs, MIs, MIIs, Pos, ER, and Ps), which is in line with previous evolutionary classification of *HSP20* genes in *Arabidopsis* and rice (Scharf et al., 2001; Ouyang et al., 2009), indicating a close relationship among *HSP20* genes from apple, *Arabidopsis* and rice. In addition, most apple *HSP20* genes were classified into CI-CVI, indicating that cytoplasm could be the primary functional area of the *HSP20* family in apple. Gene structure has been documented to function directly in the evolution of multiple gene families (Ji et al., 2019). Gene structure analysis indicated that most apple *HSP20* genes have no introns (60.9%) or one intron (31.7%), suggesting relatively simple gene structures. Similarly, most (93.8%) grape *HSP20* genes have no introns or one short intron (Ji et al., 2019).

Genes with few or no introns are considered to be rapidly activated in response to various stresses (Jeffares et al., 2008). In our study, most apple *HSP20* genes were rapidly induced after 4 h of heat stress, which may support the rapid response. To more comprehensively investigate the evolution of *HSP20* genes, the encoded conserved motifs were also studied. Our results showed that most of the apple *HSP20* genes had five to seven conserved motifs and almost all the *HSP20* genes contained motif 1. This indicates a slow evolutionary rate. Furthermore, we found that most *HSP20* genes in the same subfamilies showed conserved motifs and similar exon/intron structures, supporting their close evolutionary relationship and the classification of subfamilies. Genes in the same subfamily tends to share similar motif and exon–intron organization, which was also reported in tomato (Yu et al., 2016).

*Cis*-elements in the promoters of genes have been documented as essential in plant physiological response and environmental stress (Yamaguchi-Shinozaki and Shinozaki, 2005). We identified *cis-*elements in the putative promoter regions of apple *HSP20* genes. Numerous hormone responsive, stress-responsive and plant development-related *cis*-elements were found. Among these *cis*-elements, the hormone responsive elements accounted for the highest proportion. Most *HSP20* genes contained stress-related response elements. The present results suggest that most apple *HSP20* genes might be significantly related to stress response. Similar regulatory patterns of *HSP20* genes were also found in pepper and grape (Guo et al., 2015; Ji et al., 2019). In addition, all apple *HSP20* genes contain light signal response elements, which indicate that *HSP20s* were essential in plant growth and development.

Previous studies have revealed that *HSP20s* function directly in plant responses to various stresses (Waters et al., 1996; Guo et al., 2015; Zhao et al., 2018; He et al., 2019; Ji et al., 2019). In this study, the expression profiles of apple *HSP20* genes under heat stress revealed that the apple *HSP20* genes are involved in heat response. qRT-PCR analysis indicated that most apple *HSP20* genes were up-regulated under heat stress. It is interesting to note that the relative expression levels of 12 *HSP20* genes (*HSP20-8, 13, 16, 17, 18, 19, 23, 29, 35, 36, 37*, and *38*) were extremely up-regulated after 4 h of heat stress. The results from this study suggest that these genes might be mainly involved in the heat stress biological pathway. Similarly, most of the pepper and potato *HSP20s* were also up-regulated in response to heat stress (Guo et al., 2015; Zhao et al., 2018).

Transgenic research has demonstrated the positive role of *HSP20* genes in responding to heat, such as *WsHSP26* in *Arabidopsis* (Mu et al., 2013), and *OsHsp17.7* and *OsHSP20* in rice (Murakami et al., 2004; Guo et al., 2020). In addition, some *HSP20s* showed the same expression profile in response to heat stress, being upregulated with peak expression levels at 4 h, suggesting that these *HSP20s* were co-expressed in response to heat stress. Furthermore, Guo et al. (2015) found that the inducibilities of *HSP20* genes in response to heat stress were obviously different in pepper with different tolerance. Collectively, these results indicate that *HSP20* genes may be positively involved in heat stress responses in plants. Induction of *HSP20* genes by heat stress is well-known (Waters et al., 1996). However, some heat-regulated genes were barely expressed or downregulated in response to cold stress, which indicated that *HSP20* genes were negatively or only slightly involved in the response to cold stress. These results imply that the signaling pathways in plant response to heat and cold stress might be different.

## Conclusion

This study identified 41 *HSP20* genes in apple. These genes are unequally distributed on 15 chromosomes and were classified into 10 subfamilies based on the phylogenetic tree and subcellular localization. The basic classification, genome distribution, gene structures, conserved motifs, and *cis* acting elements of these genes were analyzed, which will be helpful for a better understanding of the evolutionary relationships of the *HSP20* gene family. Transcriptome analysis revealed that most apple *HSP20* genes were highly induced by heat stress, whereas these genes were nearly unchanged or downregulated under cold stress, indicating that *HSP20* genes were positively involved in heat stress responses in apple. Additionally, we identified several *HSP20* genes that may be utilized as candidates for improving heat stress tolerance. The results presented here will lay a solid foundation for functional characterization of *HSP20* genes through gene-transfer techniques to improve heat tolerance of apple.

## Data Availability Statement

The datasets presented in this study can be found in online repositories. The names of the repository/repositories and accession number(s) can be found in the article/Supplementary Material.

## Author Contributions

TB designed the experiment, analyzed the data, and drafted the manuscript. FY, CS, and HW collected the public dataset and performed bioinformatics analysis. XZ and SS analyzed the data. JJ and MW assisted with revisions to the manuscript. All authors have read and agreed to the published version of the manuscript.

## Conflict of Interest

The authors declare that the research was conducted in the absence of any commercial or financial relationships that could be construed as a potential conflict of interest.

## Footnotes

1 https://www.rosaceae.org/

2 http://pfam.xfam.org

3 http://smart.embl-heidelberg.de/

4 https://web.expasy.org/protparam/

5 http://linux1.softberry.com/

6 http://plants.ensembl.org/index.html

7 http://www.megasoftware.net/

8 http://meme-suite.org/tools/meme

9 http://bioinformatics.psb.ugent.be/webtools/plantcare/html/

10 http://circos.ca/

11 https://iris.angers.inra.fr/gddh13/the-apple-genome-downloads.html

12 https://www.ncbi.nlm.nih.gov/sra/PRJNA665791

## Supplementary Material

Supplementary Table 1 qRT-PCR primers used in this study.

Supplementary Table 2 The CDS sequences, deduced amino acid and promoter sequences of apple *HSP20* genes.

Supplementary Table 3 Information of *HSP20* genes from apple, rice and *Arabidopsis.*

Supplementary Table 4 List of the putative motifs of HSP20 proteins.

Supplementary Table 5 Tandem and segmental duplicated *HSP20* gene pairs within apple genome.

Supplementary Table 6 Expression levels for selected apple *HSP20* genes under heat and cold stress.
