# Supplementary material for: Genome-Wide Characterization of the HSP20 Gene Family Identifies Potential Members Involved in Temperature Stress Response in Apple
Source: Front Genet. 2020 Nov 6;11:609184. doi: 10.3389/fgene.2020.609184 (PMC7678413; doi:10.3389/fgene.2020.609184)
Supplement: Supplementary Table 4 — List of the putative motifs of HSP20 proteins. [file Table_4.DOCX]

**Table S4** List of the putative motifs of HSP20 proteins

| **Motif** | **Length (AA)** | **Sequence** |
| --- | --- | --- |
| Motif1 | 29 | VERSSGKFLRKFRLPENAKVDQIKAEMEB |
| Motif2 | 16 | DVPGLKKEDVKVEVED |
| Motif3 | 21 | AKAMANTPADWKETPNAHVFV |
| Motif4 | 15 | GVLTVTVPKLPPPKP |
| Motif5 | 15 | RVLQISGERKREEEE |
| Motif6 | 15 | FDPFSLBLWDPFEDF |
| Motif7 | 24 | LFSTLQDILDAADDAEKSSNAPTS |
| Motif8 | 6 | GDKWHR |
| Motif9 | 50 | QRQZARPRKLAAPAPPIGLLDPFSPVRTLRQMLDTVDRLMEDPVAYPGRG |
| Motif10 | 21 | YGEGRAPWDIKDGEHEYKMRF |
